# Supplementary material for: Sports instructors’ job insecurity and turnover intention in South Korea: A moderated mediation model of abusive supervision, work engagement, and perceived organizational support
Source: PLoS One. 2026 Apr 28;21(4):e0347639. doi: 10.1371/journal.pone.0347639 (PMC13123942; doi:10.1371/journal.pone.0347639)
Supplement: Attachment — Submitted filename: Response to Reviewers.docx [file pone.0347639.s003.docx]

**Journal Requirements**

**Review 1.**

Please ensure that your manuscript meets PLOS ONE's style requirements, including those for file naming. The PLOS ONE style templates can be found at
https://journals.plos.org/plosone/s/file?id=wjVg/PLOSOne_formatting_sample_main_body.pdf and
https://journals.plos.org/plosone/s/file?id=ba62/PLOSOne_formatting_sample_title_authors_affiliations.pdf

**Response 1.**

We have thoroughly reviewed the file and made the necessary revisions in strict accordance with the journal’s formatting guidelines. We would appreciate it if you could kindly review the revised version.

**Review 2**

In the online submission form, you indicated that further inquiries beyond the data presented in this paper can be directed to the corresponding authors.
All PLOS journals now require all data underlying the findings described in their manuscript to be freely available to other researchers, either 1. In a public repository, 2. Within the manuscript itself, or 3. Uploaded as supplementary information.
This policy applies to all data except where public deposition would breach compliance with the protocol approved by your research ethics board. If your data cannot be made publicly available for ethical or legal reasons (e.g., public availability would compromise patient privacy), please explain your reasons on resubmission and your exemption request will be escalated for approval.

**Response 2.**

All raw data underlying the findings described in this manuscript are publicly available in the Open Science Framework (OSF) repository and can be accessed at <https://osf.io/92wsf/>.

**Review 3.**

When completing the data availability statement of the submission form, you indicated that you will make your data available on acceptance. We strongly recommend all authors decide on a data sharing plan before acceptance, as the process can be lengthy and hold up publication timelines. Please note that, though access restrictions are acceptable now, your entire data will need to be made freely accessible if your manuscript is accepted for publication. This policy applies to all data except where public deposition would breach compliance with the protocol approved by your research ethics board. If you are unable to adhere to our open data policy, please kindly revise your statement to explain your reasoning and we will seek the editor's input on an exemption. Please be assured that, once you have provided your new statement, the assessment of your exemption will not hold up the peer review process.

**Response 3.**

Thank you for your message and for the clarification regarding PLOS’s data availability policy.

In our original submission, we indicated that the data would be made available upon acceptance. However, we have now deposited the raw data underlying the findings of this study in the Open Science Framework (OSF) repository. The data are freely and publicly accessible at the following URL: https://osf.io/92wsf/.

We confirm that making these data publicly available does not breach the protocol approved by our institutional review board. We will update the Data Availability Statement in the submission system accordingly.

**Review 4.**

Your ethics statement should only appear in the Methods section of your manuscript. If your ethics statement is written in any section besides the Methods, please move it to the Methods section and delete it from any other section. Please ensure that your ethics statement is included in your manuscript, as the ethics statement entered into the online submission form will not be published alongside your manuscript.

**Response 4.**

We appreciate the reviewer’s careful attention to the ethical reporting standards. In response to this comment, we have revised the manuscript to ensure that the ethics statement is clearly presented within the Methods section, specifically under the “Participants and Procedure” subsection.

The following statement has been added to the end of the “Participants and Procedure” section:

*This study was approved by the Institutional Review Board of a public institution designated by the Ministry of Health and Welfare of Korea (Approval No. P01-202504-01-007). All participants provided informed consent prior to participation, and no identifiable personal information was collected.*

In addition, we have carefully reviewed the manuscript to ensure consistency in ethical reporting. The ethics-related information remains appropriately included in the end-of-manuscript sections (e.g., Institutional Review Board Statement and Informed Consent Statement) in accordance with journal guidelines.

**Review 5.**

If the reviewer comments include a recommendation to cite specific previously published works, please review and evaluate these publications to determine whether they are relevant and should be cited. There is no requirement to cite these works unless the editor has indicated otherwise.

**Response 5.**

We appreciate the reviewer’s suggestion to consider additional references. We have carefully reviewed the recommended publications and evaluated their relevance to the present study. Relevant references have been incorporated into the revised manuscript where appropriate, while those deemed less directly related were not included to maintain the focus and coherence of the study.

**Reviewer 1.**

**Review 1.**
While the study has potential, major revisions are required:
These relationships are well documented in organizational psychology literature.

The authors should pay attention to clarify:
What gap in sport management literature this study fills?
Why non-regular sports instructors represent a theoretically meaningful case?
How findings refine JD-R or COR theory in precarious employment contexts?

**Response 1.**

We appreciate the reviewer’s insightful suggestion to strengthen the theoretical and contextual positioning of this study. In response, we have substantially revised the Introduction section to clarify both the research gap and the contextual relevance.

First, we added a new paragraph in the final part of the Introduction (immediately before the study purpose) to explicitly highlight the theoretical gap. While prior research has examined job insecurity across general occupational sectors, limited attention has been given to its complex mechanisms within the sports management context. In particular, we emphasize that this study extends the literature by examining a moderated mediation model incorporating abusive supervision, work engagement, and perceived organizational support among non-regular sports instructors.

Second, we further elaborated on the theoretical relevance of non-regular sports instructors as a focal population. We clarified that their employment conditions—such as contract-based work, performance uncertainty, and weak organizational attachment—make them a meaningful context for extending the Job Demands–Resources (JD-R) model and Conservation of Resources (COR) theory in precarious employment settings.

Finally, we incorporated a discussion of the South Korean labor market context, particularly the widespread use of non-regular employment and its structural implications for job insecurity and employee well-being. This addition strengthens the contextual grounding of the study and highlights its relevance for understanding employment precarity in institutional settings.

**Review 2.**

The authors should explain why some paths are insignificant in SEM but significant in PROCESS? For instance, AS ⎝ TI is insignificant (β = 0.095, p = 0.108) in SEM but it significantly predicted TI (β = 0.253, p = 0.003).

**Response 2**

Thank you for this valuable comment. We have added an explanation in the Discussion section. SEM estimates relationships simultaneously using latent variables while accounting for measurement error, whereas PROCESS relies on observed variables with OLS regression and bootstrapping. Due to these methodological differences, discrepancies in statistical significance may occur. This clarification has been incorporated into the revised manuscript.

**Review 3.**

Some claims need to be checked. For instance, “Individuals with high work engagement tended to express stronger desires to transition into better job positions” contradicts the buffering hypothesis and your statistical results. These interpretations appear to misread conditional indirect effects.

**Response 3**

We thank the reviewer for this important and constructive comment. We agree that the original wording could be interpreted as inconsistent with both our buffering hypothesis and the observed conditional indirect effects. In response, we carefully revised the relevant parts of the Discussion section to ensure that the interpretation is fully aligned with the statistical results.

Specifically, we removed statements suggesting that individuals with high work engagement or high perceived organizational support were more likely to seek better job positions. These sentences could misrepresent the direction of the moderated mediation effects. We replaced them with a more accurate interpretation indicating that the indirect effect of job insecurity on turnover intention through abusive supervision was stronger at lower levels of work engagement and perceived organizational support, and weaker at higher levels of these resources.

Accordingly, the revised text now clarifies that both work engagement and perceived organizational support function as buffering resources that attenuate the negative indirect pathway from job insecurity to turnover intention via abusive supervision. These revisions improve the consistency between the hypotheses, statistical findings, and theoretical interpretation.

**Review 4.**

Strong causal wording should be softened.
The manuscript should explicitly acknowledge sampling bias and limits to external validity.
The manuscript needs English editing.
More robustness checks should be conducted.

**Response 4.**

Thank you for these constructive suggestions. In response, we revised the manuscript in four ways. First, we softened causal wording throughout the Introduction, Theoretical Background, and Hypothesis sections to ensure that the language is consistent with the cross-sectional design. Second, we added explicit statements on sampling bias and limited external validity in the Methods and Discussion sections. Third, we thoroughly edited the manuscript for clarity, concision, and overall English flow. Fourth, we added robustness-check procedures to the Data Analysis section and reported the stability of the findings in the Results section.

**Reviewer 2.**

**Review 1.**

The flow and style of writing can be reviewed to make it more crisp. Conduct a thorough proofread.

**Response 1.**

Thank you for these constructive suggestions. In response, we revised the manuscript in three ways. First, we softened causal wording throughout the Introduction, Theoretical Background, and Hypothesis sections to ensure that the language is consistent with the cross-sectional design. Second, we added explicit statements on sampling bias and limited external validity in the Methods and Discussion sections. Third, we thoroughly edited the manuscript for clarity, concision, and overall English flow.

**Review 2.**

Standardize to "abusive supervision" throughout, as it aligns with the literature (e.g., Tepper, 2000; Mackey et al., 2017). If "impersonal" is intentional, define it clearly and justify the distinction.

**Response 2.** I have checked and corrected all the incorrect words. Thank you.

**Review 3.**

Strengthen the gap analysis. While prior studies examine job insecurity in general sectors, this is among the first to model moderated mediation in sports instructors. Add a paragraph on South Korean context (e.g., labor laws affecting non-regular workers).

**Response 3.**

Thank you for this valuable comment. In response, we revised the Introduction section by adding a new gap-analysis paragraph immediately before the study purpose paragraph. Specifically, we clarified that, although prior studies have examined job insecurity in general occupational sectors, limited research has investigated its moderated mediation mechanism within the sport management context, particularly among sports instructors. We also emphasized that non-regular sports instructors represent a theoretically meaningful case because their work is characterized by close supervisory dependence, emotional labor, and unstable employment arrangements. In addition, we incorporated the South Korean labor context to highlight how the widespread use of non-regular employment and related structural inequalities in job security and welfare benefits make this setting particularly relevant for examining precarious employment. Because the institutional details were already discussed in the Literature Review, the Introduction was revised to provide a concise contextual justification while avoiding redundancy. These revisions strengthen the theoretical positioning and contextual relevance of the study.

**Review 4.**

Ensure all tables/figures are self-explanatory with full captions. Report effect sizes (e.g., f² for moderation). If multicollinearity is a concern (from correlations in Table 3), mention VIF checks.

**Response 4.**

All tables and figures have been revised to include clearer notes and definitions of abbreviations. Additionally, effect sizes (Cohen’s f²) were calculated and reported for moderation effects, indicating small-to-moderate magnitudes.
We also assessed multicollinearity using variance inflation factor (VIF), and all values were below the recommended threshold, indicating no multicollinearity concerns.

**Review 5.**

Add a dedicated "Measures" subsection listing instruments (e.g., "Job insecurity was measured using Sverke et al.'s (2002) 4-item scale").

Discuss common method bias (e.g., via Harman's test) since all data are self-reported and cross-sectional.

Acknowledge limitations of convenience sampling in reducing generalizability.

**Response 5.**

Thank you for this valuable suggestion. We have revised the manuscript accordingly.

First, a dedicated "Measures" subsection has been added to clearly specify the measurement instruments used for each construct, including their original sources and number of items.

Second, to address potential common method bias due to the use of self-reported and cross-sectional data, Harman’s single-factor test was conducted. The results indicated that no single factor accounted for the majority of the variance, suggesting that common method bias is unlikely to be a serious concern.

Finally, we have explicitly acknowledged the limitation of convenience sampling in the Discussion section, noting that it may restrict the generalizability of the findings.

These revisions have been incorporated into the manuscript.

**Review 6.**

Some citations have future dates (e.g., Guo et al., 2025; Jung et al., 2024), which may indicate in-press works. Ensure these are updated if published.

**Response 6.**

This journal has been removed as it was deemed problematic.
